# Pregnancy; an opportunity to return to a healthy lifestyle: a qualitative study

**DOI:** 10.1186/s12884-021-04213-6

**Published:** 2021-11-05

**Authors:** Razieh Bagherzadeh, Tayebeh Gharibi, Bahare Safavi, Seyyedeh Zahra Mohammadi, Fatemeh Karami, Sedigheh Keshavarz

**Affiliations:** 1 Department of Midwifery, School of Nursing and Midwifery, Bushehr University of Medical Sciences, Bushehr, Iran; 2 Nursing and Midwifery Faculty, Bushehr University of Medical Sciences, Bushehr, Iran; 3 School of Nursing and Midwifery, Bushehr University of Medical Sciences, Bushehr, Iran; 4 Health Center of Midwifery, Bushehr University of Medical Sciences, Bushehr, Iran; 5 Baqiyatallah Azam Hospital, Maternity Ward, Bushehr University of Medical Sciences, Bushehr, Iran; 6 Department of Midwifery, School of Nursing and Midwifery, Bushehr University of Medical Sciences, Bushehr, Iran

**Keywords:** Pregnancy, Healthy Lifestyle, Postpartum

## Abstract

**Background:**

The lifestyle of the mother during pregnancy can affectthe health of their baby. Since lifestyle change is a sociocultural act and the motivations associated with lifestyle patterns during pregnancy cannot be explained in quantitative studies, a comprehensive study of the lifestyle during pregnancy and factors influencing its patterns was needed to investigate it from different aspects. Thus, the present study aimed to explore ‘mothers’ perceptions and experiences about lifestyle patterns during and after pregnancy and the reasons for adopting these lifestyles.

**Methods:**

The present study, conducted on 20 pregnant or postpartum women living in Bushehr, Iran, has used a conventional content analysis approach.

The purposeful sampling method was used with maximum diversity and continued until data saturation. data were collected through face-to-face, in-depth, semi-structured interviews. Informed consent was obtained from all participants, and assuringthe confidentiality of their information. MAXQDA 10 software was used to analyze the data.

**Results:**

Four main themes were defined after data analysis; "Being a mother as motivation for adopting a new healthy lifestyle"; "Access to information from media and supports from physicians as facilitators of adopting healthy lifestyle"; "Aspects of lifestyle modifications" and "Durability of healthy lifestyles"**.** When women become pregnant, they feel a responsibility tohave a healthy pregnancy. They care about their fetuses more than themselves, which motivated them to look for the best lifestyle. In this way, access information from mass media and recommendations from professionals (physicians, midwives, and other health care providers) were helpful factors to have a healthy lifestyle, leading to modifying physical, mental, and religious aspects of lifestyle. However, despite reminding the advantages of a healthy lifestyle, these changesshift to a pre-pregnancy lifestyle due to the cessation of support and care provided during pregnancy.

**Conclusion:**

The study results showed that pregnant women should be motivated to modify their lifestyle andadopt healthy lifestyles. Pregnant women seek to modify their lifestyle because of motherhood responsibility and and having a healthy baby. Access to information and supports from various sources promote a mother’s inner decision to change, leading to modifying different aspects of life. However, these modifications often shift to the pre-pregnancy lifestyle due to cessation of supports and care, despite reminding the benefits of the lifestyle change.

Health care providers should consider supportive measures during pregnancy and postpartum.

## Background

Pregnancy, as a critical stage, affect a human’s life profoundly [1]. Pregnancy is a potentially life-changing phenomemon opening up new responsibilities, joys, and concerns for women [2]. The mother’s health during pregnancy can directly affect the baby’s health [3, 4]. Pregnancy changesmother’s physical and mental balances, as well as her health behaviors and lifestyle. Lifestyle in pregnancy can have enduring effects on the mother and baby’s health [5]. The term lifestyle, first described by Alfred Adler, is often used to express the individual’s lifestyle and reflects a wide range of values, beliefs, and social activities and affects health. Other aspects affecting the lifestyle include controlling nutrition, doing exercise, self-care, quitting smoking, alcohol and illicit drugs, having social relationships, and controlling stress [6]. Lifestyle covers all of one’s actions and behaviors, interactions with others, nature, and generally the social environment him/ her that is usually tangible, describable, and measurable [7]. The social, medical, and environmental conditions ( such as physical and mental state, genetic disorders, teratogenic agents ) can dramatically affect pregnancy. Studies on lifestyle during pregnancy have only addressed some aspects of lifestyle and its effect on the fetus and baby. For example, one Meta-analysis showed a decrease in smoking, consuming alcohol, and obesity during preconception care and pregnancy [8].

According to the study of Lindqvist et al.’s study; (2017), most pregnant women reported that they wanted to increase their physical activity, improve their dietary habits, and lose their weight. They estimated that their ability to change their lifestyle habits equals to their motivation for change [9]. Some studies reported that poor diet during pregnancy is associated with weight gain and a higher risk of adverse maternal and neonatal outcomes, including hypertension in pregnancy, gestational diabetes, miscarriage, stillbirth, preterm birth, neonatal mortality, and inadequate fetal development. Healthy eating habit before or during pregnancy was associated with a lower riof hypertension and postpartum depression. Further, the impact of lifestyle on the child's neurological development is demonstrated [8, 10–14]. Some studies examined the impact of couple interactions on pregnancy. Accordingly, Urquia et al. (2017) showed that lack of cooperation and commitment of the partners during pregnancy is associated with maternal and neonatal outcomes [15]. Some studies addressed the effects of dietary intake, tobacco use, substance abuse, and stress during pregnancy on the mother and the fetus [16–18]. Some studies on lifestyle changes showed mothers’ decision to lifestyle promotion [19]. Different studies investigated the impact of educational interventions on lifestyle [20]. For example, Holton et al.’s study results (2017) showed that obese women would benefit from additional information and support regarding weight management during preconception care, pregnancy, and the postpartum period [21]. A study by Jinguo et al. (2020) showed that midwife-led prenatal education and the provision of relevant evidence-based resources had a positive effect. This education helped transform health ‘providers’ attitudes towards education about lifestyle during pregnancy from a passive routine ‘must do’ tasks to an active process with a focus on healthy lifestyle and engagement of pregnant women [22]. Performed studies about lifestyle in pregnancy are quantitative and focused on some aspect of lifestyles such as obesity, weight change, or exercise [23, 24]. However, little attention has been paid to lifestyle changes during pregnancy and and motivations. Since lifestyle modifications are a sociocultural issue and the motivations during pregnancy cannot be explained in quantitative studies, a comprehensive study on the lifestyle during pregnancy and factors influencing its change was needed to investigate this issue from different aspects. It can entice researchers to do further studies and plan to improve maternal health. Therefore, the present study aimed to explore mothers' perceptions and experiences regarding the lifestyle patterns during and after pregnancy and the reasons for adopting these lifestyles.

## Method

The present study used a qualitative content analysis with a conventional approach. It aimed to investigate participants’ experiences about lifestyle patterns during pregnancy and their contributing factors. Qualitative content analysis is a research method for the subjective interpretation of textual data content through the processes of systematic classification, coding, and thematic design or design of known patterns [25]. The study population consisted of all pregnant or postpartum women in Bushehr, Iran. Inclusion criteria included postpartum (6-10 hours after delivery) or pregnant women in the third trimester (week 28 and over); ability to speak in Farsi language; and willingness to participate in the study. Unwillingness to participate in the interview was the exclusion criteria. After obtaining permission from the Vice Chancellor for Research of Bushehr University of Medical Sciences, the interviewed started by a female with Ph.D. in Reproductive Health, 25 years of experience in midwifery and a faculty member of the midwifery department (First author). From the beginning through the completion of data collection, she met 90 pregnant or postpartum women in the Prenatal Care Centers and the Postpartum ward of the Shohadaye-e Khalij-e-Fars and Salman Farsi hospitals in Bushehr. Sixty women were eligible based on the inclusion criteria. Since the study aimed to reach a wide range of different perspectives, purposeful sampling was used with maximum variation in age, occupations , level of education, economic status, and the frequency of pregnancies. Sampling was performed until the saturation of data, i.e. the researcher felt no new data would be obtained and the obtained data were repeating the previous data [26]. Data saturation was attained by interview #18, but two additional interviews were conducted for assurance. Twenty participants included in the study, of whom five were interviewed twice; so, a total of 25 interviews were performed. Two participants were interviewed with consent, then regretted that their audio files were deleted in their presence. One participant called for the deletion of the interview during the coding phase and was excluded from the analysis process.

The interview was performed in a completely confidentially. No one other than the interviewer and the participant was present during the interview. Initially, the researcher introduced herself to the participants. She explained her professional experiences and the importance and objective of the study, then obtained informed consent from participants in the study for audio recordings. Face-to-face interviews were performed in the postpartum ward of hospitals and prenatal care centers by the participants’ agreement. A semi-structured interview guide (Supplement 1 and 2) was formulated by the research team and expanded after initial interviews; for example, “Has your lifestyle changed during pregnancy?”, “What are your lifestyle changes?”, and “What caused these changes?” This interview guide was utilized in all interviews. The sequence of questions was not the same for all participants; the questions and answeres were discussed depending on the interview process or the interviewee. Each interview lasted 30-60 minutes, depending on the tolerance and willingness of the participant. All interviews were audio-taped. At the end of each interview, the participants were asked to explain any remaining issues; then, a contact number was taken from all participants to request a re-interview, if necessary.

The Graneheim & Lundman method and MAXQDA software (version 10) were used to analyze the data [27] manage data, respectively. For this purpose, immediately after each interview, recordings were transcribed using the Word Processing software 2010 (in Farsi language) to obtain a transparent mode of the participants' thoughts, behaviors, ideas, and experiences. Non-verbal messages of participants, such as tone of speech, silence, emphasis, etc., were noted in the transcribed text. The whole of each interview was considered as the unit of analysis. Graneheim and Lundman (2004) [27] pointed out that the most suitable unit of analysis is whole interviews or observational protocols that are large enough to be considered whole and small enough to be kept in mind as a context for meaning unit during the analysis process.

Before coding, the text was thoroughly read several times to be fully familiar with the data. Afterward, meaning units were identified and coded. The meaning unit included the words, sentences, or paragraphs based on the context. The label of a meaning unit formed a code, highlighting the precise words from the text that appeared to capture key concepts or viewpoints. Next, the researcher made notes from her first impressions, thoughts, and initial analysis. Labels for codes emerged after the accomplisgment of this process. Then, codes were sorted into subcategories based on their relationship. A large numbers of subcategories were organized into a smaller number of heterogeneous internally homogeneous and externally heterogeneous categories, and then, the organized categories formed themes.

The information accuracy was examined using the Lincoln & Guba method (cited by Toubine & Begley, 2004). They specified their concept of trustworthiness by introducing criteria of Credibility, Transferability, Dependability and Confirmability. Credibility addresses the issue of “'fit' between 'participants' views and the ' 'researcher's representation. Transferability refers to the generalizability of inquiry. Dependability is achieved through a process of auditing. Confirmability suggeststhat data and interpretations of the findings are not figments of the ' 'inquirer’s imagination but are derived from the data [28].

Effective communication with the participants, long-term involvement, and complete immersion in the data were considered to increase the validity of the data. The sameame interviewer cinducted all interviewes. The findings were checked with the participants. The interview and the codes were sent to 10 of the interviewees (four in-person and six via e-mail). Two participants rejected three codes altogether as they meantsomething else, then modified by the explanations given. Two authors with high consistency coded interviewes simultaneously. Controversies were settled, and a consensus was obtained on coding. Another two authors (The first and fourth authors) evaluated categories, subcategories, and the relevant codes and reach a consensus after discussing the naming of categories and subcategories. Subcategories were linked and classified by codes and semantic units. To increase the acceptability of the data, all codes and categories were assessed by a qualified expert in reproductive health that not involved in the research team. Accordingly, the names of two categories were changed after the author's consensus.

The Ethics committee of Bushehr University of Medical Sciences approved the study protocol (ethical code: IR.BPUMS.REC.1397.082). Ethical issues, include taking informed consent to participate in the study and recording interviews,. Participants were assured about confidentiality of information and the deletion of audio files after completing the work. Written informed consent was obtained from all patients.

## Results

Participants were in the 18-to-40 age range. ; seven participants were pregnant or primiparous, and 12 were multiparous. Table 1 shows demographic characteristics of the participants. Four themes, ten categories, and 28 subcategories were obtained from the analysis of the interviews (Table 2).

**Table 1.** Demographic characteristics of the study participants

Variable	Category of variable	Number
**Educational levels of participants (frequency)**	Elementary school	1
	Incomplete high school diploma	4
	High school diploma	9
	Associate degree	2
	' 'Bachelor’s degree	1
	Master degree	2
	PhD	1
**Educational levels of partners**	Incomplete high school diploma	3
	High school diploma	8
	Associate degree	1
	' 'Bachelor’s degree	5
	Master degree	2
	PhD	1
**Live birth**	No children	2
	Had a child	9
	Had two children	5
	Had three children	3
	Had four children	1
**Time of interview**	The third trimester of gestation	9
	After delivery	11

**Table 2.** Themes, main categories, and subcategories describing mothers’ perceptions and experiences regarding the lifestyle patterns during and after pregnancy and the reasons for adopting these lifestyles

Theme	Main category	Subcategory
**Being a mother as main motivation for adopting a healthy lifestyle**	Sens of motherhood as motivation for adopting healthy life style	Motherhood as a path to perfection
		responsibility to complete the pregnancy in the best way
	Baby as the first priority for mother motivated them to adopt a healthy lifestyle	Provide a safe environment for their fetus
		Fighting harmful habits for the health of the fetus
**Access to information and supports as facilitators to adopt a healthy lifestyle**	Access information from mass media	Information from TV
		Information from Internet
		Information from other source of media
	Support from health care provider	Support from Physician
		Support from midwife
		Support from other health care provider
	Support from family and friends	' 'grandmother’s support
		' 'Partner’s support
		Other ‘relatives’ support
		‘Friends’ support
**Aspects of life style modifications**	Physical	Change in diet
		Improved sleep habits
		Observing hygiene and breaking harmful habits
		Marital relationship
	Mental	Trying to create inner peace
		Reduced arguments with partner
	Religious	Feel close to God
		Following religious orders
**Durability of healthy life style**	Striving for sustainability of healthy life style	Remembering the benefits of healthy life style
		Future of motherhood
	Causes of lifestyle changes instability	Cessation of supports
		Limitation of time and endurance
		Return to work
		Reduced health care

### Being a mother as the main motivation for adopting a healthy lifestyle

This theme included two categories of “sense of motherhood as the main motivation for adopting healthy lifestyle”, and “baby as the priority for mother entice her to adopt a healthy lifestyle”. Most participants stated that when they got pregnant, they decided to change or improve their lifestyle and adopt a healthy lifestyle because of the sense of motherhood. Mothers feel they are following the path to perfection. Therefore, they took a responsibility of having the healthiest pregnancy. Theyprioritize their baby that motivates them to look for the best lifestyle, thus providing a healthy setting for their fetus.

Two participants expressed their sense of motherhood and the battle of motherhood for their ' 'children’s health as follows:

Pregnancy seems to be a path to perfection for a woman. The pregnancy should have gone in the best possible way. I felt responsible for having a safe pregnancy (Gravid 1; Ph.D.); "I had to change my bad habits. I was not alone. I did not want to hurt my baby. It was like a battle" (Gravid 1; Diploma); “You know, as soon as the pregnancy test result is positive, your senses change. It is something strange. You are ready to do everything for your baby” (Gravid 3; Diploma).

Some women called quitting unhealthy behaviors a battle, which is difficult or even impossible to win. One of the participants commented on her experience: "Sometimes it is difficult; very difficult. I was 22 when I got pregnant with my first baby. I had fun; I used to smoke fruit-flavored hookah, although they were saying it is not good for the baby. I was in agony! I fought. First, I couldn't afford it; I was quitting smoking for a week and starting again, finally, I could overcome it because I want to have a healthy baby "(Gravid-3; Bachelor's degree).

### Access to information and supports as facilitators to adopt a healthy lifestyle

This theme included three categories of “access information from mass media”, “support from the health care provider”, and “Support from family and friends”.

Access to information and supports, including recommendations and support from physicians, midwives, and other health care providers was another factor in making the right lifestyle choice. A participant added:

“If someone wants to live a healthy life- I mean a healthy lifestyle- well, the context is provided! The doctor gives you information, more or less; going to health centers, the midwife tells you what to do; TV and media advises. You will find many things by surfing the net if you can. I search everything I want, even a recipe, on the internet” (Gravid-4; Master's degree).

Supports from relatives and friends facilitated lifestyle changes. Support from the mother was significant. Participants cited the role of the mother, or the future grandmother is beyond a usual support. They noted that their mothers’ experiences helped them better make sense of motherhood and choose the appropriate lifestyle in pregnancy. They said that mothers are not only supportive but also induce a sense of motherhood.

“My mother used to say what she was doing in her pregnancy; she even said how she was feeling and all were helpful. Since I got pregnant, she feltherself a grandmother and cared about her grandchild’s health as well as my health,. Her experiences helped me a lot in how to behave, how to sleep, and all that” (Gravid-2; Associate degree).

Another participant who was pregnant with her fifth child expressed her understanding and experiences: “The mother is worthy. I don't have her anymore. It is very difficult (with crying). She was doing everything as she heard that she got pregnant. She was very supportive; always saying what to eat to be useful for myself and baby; I could not make food; she was making the best. When I was pregnant with my third child, she took care of my two otherchildren to give me a break. It was a blessing to tell me what to do. I thought she would always be here; I didn't keep her experiences in my mind as I was sure she would be here with her experiences in the next pregnancy; but now I am full of regret” (Gravid-5; Diploma).

Participants cited the partner and other relatives support as a contributing factor to lifestyle modifications. Some participants attributed half of the changes to the behavior of the partner. Some acknowledged that their partners change their behaviors during their pregnancies, which helps them adopt the proper lifestyle. Two participants noted that partner's misconduct in pregnancy made them unable to strive for a healthy pregnancy. One participant stated that her husband was supportive of previous pregnancies but gave very little support in the current pregnancy because he did not want more children.

“My husband is fine. He helps a lot during pregnancy. He tries everything that goes well and keeps me in peace. And that helps me get through pregnancy more easily”. (Gravid-2; Diploma).

“Now the pregnancy is closing the due date, and it is about to delivery; Thank God! I lived the way I was before; too bad. I wishd not toget pregnant. My husband would bitter my life; he never asks how I am, whether I need help or not, but always gets on my nerves and always ignores me. May God watch over my baby. I had the same experience with my previous pregnancy, I wished not to have unhealthy eating habits during pregnancy, not even smoke hookah, but it is impossible” (Gravid-3; Diploma).

“My husband is not always fine and calm, but in previous pregnancies, as soon as he knew I was pregnant, he started changing. It would help me have a delivery in peace and choose the right lifestyle.. However, it is not like ever in this pregnancy because he did not want more children. Now he does not help which makes me relaxed.. I feel the baby is not important to him; so I cannot change my way” (Gravid-3; Bachelor’s degree).

### Aspects of lifestyle modifications

Another theme extracted from the compressed information included modification in physical, mental, and religious aspects of lifestyle. The physical aspect modification consists diet changes, sleep patterns, marital sexual relationships, and quitting harmful habits. The mental aspect modification category included subcategories to create personal and family peace and follow religious orders. Some participants noted that they had chosen healthy lifestyles as much as possible throughout their lives, but most stated that they had tried to change their lifestyle in pregnancy and choose a healthy physical and mental life.

Participants’ experiences indicated that they were trying to modify pre-pregnancy eating habits that are harmful to the fetus during pregnancy. They switched to healthy foods and reduced or eliminated unhealthy foods such as fast food, chips, snacks, spices, and pickles, even striving to reduce or stop cravings for nonfoods (pica) as much as possible.

“I love sandwiches and pizzas very much, but I was eating very little during pregnancy. I would rather eat something healthier and sleep well” (Gravid-2; Diploma).

Many participants stated that as they could not take any medication during pregnancy, they tried to reduce the risk of illness by observing personal and family hygiene. Two of the participants talked about quitting hookah during pregnancy.

“I did not want to catch a cold or get poisoned. I did not want to take medicine, so I was cautious about myself and my family’s health. I maintained good hygiene. I used to smoke hookah, but not at all during pregnancy” (Gravid-2; Diploma).

Participants’ experiences indicated that they could reduce stress when they felt stressed out in the family.

“My husband and I seemed to have announced a ceasefire. I do notstart an argument. I wanted to feel relax; you know; stress is not good for the baby. I experienced my first pregnancy in peace provided by both my husband” (Gravid 3- Incomplete diploma) and I.

The experiences of some participants indicated that they felt closer to God in pregnancy. They perceived pregnancy and step-by-step embryo development as a miracle that strengthened their faith and brought them closer to God. They stated that their adherence to religious orders increased, and they were paying more attention to religious advice, especially doubting in foods. They stated that they were working harder to fulfill their obligations rather than non-pregnancy time, which made them feel more relaxed during this time.

“I was so careful of what I eat. I did not try anything in shops. I did not want to eat unhealthy things. I was also asking my husband to make Halal and pure money. My prayer was on time. I was trying to say my morning prayer on time; I gave myself a peach. Pregnancy is a miracle, and it brings you closer to God” (Gravid-4; Diploma).

### The durability of a healthy lifestyle

This theme consisted of two categories as “striving for the sustainability of healthy lifestyle” and “causes of lifestyle change instability”.

Most women who were pregnant with their first babies or were primigravida, and some multigravida stated that they decided to make positive changes to their lifestyle during pregnancy, using strategies such as remembering and reviewing the advantages of the changes, as well as striving to maintain mother’s health as a requisite for current childbearing and future pregnancies. However, most multigravida participants stated that some or all of these changes back to the pre-pregnancy time shortly after delivery. They noted discontinuation of support, especially from the partner, limited energy and time, and reduced care as the causes of lifestyle change instability. Most participants mentioned exhaustion of parenthood and lack of time as reasons for the sustainability of a healthy lifestyle. Returning to work after maternity leave was another factor that influenced the sustainability of lifestyles modifications.

Interestingly, only two of the employed participants noted thatreturning to work just after delivery was a cause for fatigue. Most employed multiparous women who back to work after delivery reported that returning isan opportunity to care for themselves because they hired a babysitter to take care of the baby when they returned to work, or the family member helped them with care. However, some argued that caring and supporting women returning to the workplace should be considered in organizational policies. Participants mentioned that untrained and inexperienced caregivers are reasons for the unsustainability of a healthy lifestyle.

“I have a healthier life now. I just want to keep fit and healthy. Motherhood is not just pregnancy; I have to raise a baby, so I have to be healthy” (Gravid 1-' 'bachelor’s degree).

“I am pregnant with my third child right now. I had a perfect lifestyle in my first pregnancy; But after delivery, I discontinue the same way after a while. My husband does not help, I feel tired; I should take care of children and clean the house” (Graevid-3; Incomplete d diploma)

“Postpartum is not like pregnancy. You are tired, you don’t have time to keep up with the things you did during pregnancy. You have no time to take a walk. Midwives even pay more attention to the baby’s weight gain and vaccination. Very few midwives ask what we are doing or what we eat” (Gravid 2- Incomplete diploma)

“Believe it or not, after my first delivery, when my maternity leave was over, and I was back to work, I could live for myself. At least I had breakfast. I take a walk to work. I wish there was a program for women who are just coming off maternity leave; a time to live for ourselvesFor example, an hour for women to have a rest at the office, if there is a place to exercise, eat food or whatever necessary for their health” (Gravid-3; Diploma).

## Discussion

The present study aimed to explore mothers' perceptions and experiences regarding the lifestyle patterns during and after pregnancy and the reasons for adopting this lifestyle. The results of the study showed that motivation is fundamental for lifestyle modifications during pregnancy. Pregnant women decide to modify their lifestyle because of feeling a sense of motherhood and and having a healthy baby. Access to information and supports from various sources promotes the mother's inner decision to change, which ultimately leads to modifying different aspects of the mother's life. However, despite reminding the advantages of a healthy lifestyle, these changes often shift to a pre-pregnancy lifestyle, especially in playing an optimal maternal role in the future, due to cessation of support and care provided during pregnancy.

Exploring the participants’ experiences showed that the sense of motherhood makes an individual change her lifestyle and battle for that change. The sense of motherhood means that the mother considers herself responsible for ending the pregnancy in the best possible way. The fetus overshadows the harmful interests of the mother, and the mother decides to replace healthy behaviors with unhealthy ones.

According to Dencker et al. (2016), the baby was the most important motivation for participants to change their eating habits and exercise during pregnancy. Although addressing only two aspects of lifestyle, the results of their study were consistent with those of the present study in terms of motivation for change [23].

Ayyala et al. (2020) on USA women showed that motivation to have a healthy baby during pregnancy and back to work after delivery were reinforcing factors for sustainable health behavior [29]. Which is inconsistent with the results of the present study. For Iranian women, the child is important than anything else. They care less about their health than the health of their children [30]. This attitude can be the reason for not referring to one's health as a motivation to change lifestyle during pregnancy.

According to Edvardsson et al. (2011), Swedish mothers were not such motivated to shift to a healthy lifestyle because they considered themselves healthy and robust, despite considering weight loss, physical activity, and healthy eating useful [31]. This finding was inconsistent with that of the present study, showing that most women needed lifestyle modifications during pregnancy. The reason for this contradiction may be different attitudes of the studied populations. Bassett-Gunter et al. (2013) concluded that attitude is the most important predictor of behavior intention [32]. Pregnant women are more motivated to change their habits and lifestyles than non-pregnant ones. However, the ability to make changes depends on the individual's motivation [9]. In the study of Edvardsson et al. (2011), the most important motivation for changing the lifestyle was , fetus health which is consistent with the results of the present study [31]. mothers who consider themselves healthy and the ones who find themselves in need to change their lifestyle have the motivation to adopt the correct behavior and distance themselves from harmful habits because of baby’s health.Pregnancy can be a good time to educate and develop health-promoting behaviors and quit harmful habits.

Staneva et al. (2016) showed that there is no correlation between maternal orientation and pregnancy distress [33]; these results are inconsistent with those of the present study, and the reason for this contradiction may be due to the study types, and different definitions of motherhood in the two studies. In the present study, however, maternity is a trigger for change that other factors (e.g. support) facilitate this change. They are not addressed in Staneva’s study.

Moreover, access to information and supports from various sources facilitated lifestyle modifications during pregnancy. Access to information was noted as an influential factor in decision-making about lifestyle change and makes mothers put into practice the optimal information gained about lifestyle change during pregnancy. The source of information may include physicians, midwives, health care providers, digital databases and mass media. Lagan et al. (2010) showed that 94% of pregnant women get information surfing on the internet and from the physician, and 83% of them made decisions accordingly [34]. Poels et al. (2017) reported that two-thirds of women seek preconception health information to prepare for pregnancy, and information they seek is related to positive changes in preconception lifestyle [35].

Considering the importance of digital databases and media as a means of accessing information, social media can provide users with false information as well.

Health care providers should identify reliable websites and introduce them to pregnant women. In addition to routine care, health care providers should allocate time to recommend a healthy lifestyle. Dencker et al. (2016) showed that a midwife, as a healthcare provider, should adhere to several principles for lifestyle change; first, give consultation without judgment and solely based on the individual’s conditions, and second, lifestyle change should be considered as a collaborative task; i e, both the midwife and the pregnant woman should participate [23].

Experiences of women indicated that changing lifestyle andreturning to a healthy lifestyle during pregnancy require the support by mothers, partners, other family members, as well as friends. Of course, the share of the future mother or grandmother was beyond just support. In addition to all-around support, participants considered the mother as a source of the sense of motherhood. In Widarsson et al.’s study (2012), the supportive role of partner, mother, and other relatives was highlighted [36] that is consistent with present study results. The absence of the mother was considered as a gap in the life of pregnant women. In Iranian culture, women often spend most of their time in their parents' home in their first pregnancy and even after childbirth to receive care for a while. Pregnant women who have lost their mothers or cannot receive maternal support should be consulted to find alternative support.

Most participants mentioned the supportive role of the partner. The results showed that women with unwanted pregnancies have little or no support from a husband. A review study found that the husband's participation and assistance during pregnancy and childbirth were useful for the husband himself and the mother and baby [37]. Lack of ' ' husband’s cooperation and commitment during pregnancy was associated with maternal and neonatal outcomes [15]. Studies showed that intimate partner violence during pregnancy could have serious health consequences for mothers and newborns, such as unwanted pregnancy, abortion, preterm labor, low birth weight, intrauterine fetal death, and like these [8, 38, 39]. Women experiencing intimate partner violence are more likely to get prenatal care late. Prenatal care visits contribute to reducing maternal and child mortality [40]. Ilska and Przybyła-Basista (2017) found that partners receiving support mediated the relationship between the prenatal concerns of women and some areas of their psychological health. Due to the support received by pregnant women from their partners, prenatal concerns stop affecting their attitude about the meaning of life, feeling of fulfillment of essential roles, and beliefs about their ability to cope with the world [41].

It seems that informing husbands about supporting their wives and their participation in prenatal care should be included in the health care plan. Likewise, setting up comprehensive care where the health care team and the pregnant woman's family are involved and trained to provide the best possible support can lead to a good pregnancy for the mother and her relatives. Studies showed that prenatal care should include both parents, but husbands are neglected in many prenatal care plans. If partners are considered in such plans and committed during pregnancy, they will be committed to childcare [36, 42]. Another important point is that the decision to change lifestyle and adopt a healthy lifestyle is made after learning about pregnancy. At the same time, the mother’s health is critical before fertilization and the early stages of fertilization and pregnancy. Unfortunately, in Iran, pre-pregnancy care is still not very important. Prenatal care and informing women and their husbands about the importance of a healthy lifestyle in preconception care should be on the agenda of health care providers.

The results showed modification in physical and psychological aspects of lifestyle and even interaction with the partner. Religious changes were also important. Most pregnant women stated that they strived to get adequate nutrition and sleep, stop harmful habits, and provide a peaceful and restful living environment during pregnancy. They stated that they tried to act in marital relations and sex to be not harmful to the fetus. This lifestyle change was more prevalent in the first pregnancy than in subsequent pregnancies, and according to some participants, these changes were subject to the support of the partner and appropriate family interactions. The study by O’Keeffe et al. (2016) on Irish women found that driving the correct behaviors during pregnancy, such as reducing or eliminating alcohol consumption and stopping smoking had increased, but a few women changed their behaviors [19]. One study found that 61% of women were satisfied with lifestyle changes in pregnancy [31]. However, the result was not comparable with those studies above due to the research method, which showed that womenwere concerned about a change in pregnancy, which should be implemented and enhanced with appropriate stimuli. Health educators can take a practical step on this path by showing the advantages of a lifestyle change for the mother and the growing fetus to the pregnant woman and the husband.

Participants stated that they felt closer to God during pregnancy and tried to follow more religious orders and avoid whatever forbidden in Islam. A study by Cyphers et al., (2017) in Eastern Pennsylvania showed that being religious is directly related to adopting health-promoting behaviors in pregnancy [43]. Iranian Muslim women believe that adhering to religious orders in pregnancy will give birth to a righteous and religious child in the future. Perhaps this is the driving factor for adopting the right lifestyle. Religious-based interventions and beliefs can promote some of the religious aspects [44].

It is worth noting that women have piritual needs besides the health-related needs.

Some reliable experts should be introduced to mothers to help them change their behavior during pregnancy.

The experiences of multiparous women indicated that they could not keep up with the changes because of reduced support and lack of time, fatigue, and stress, despite trying to maintain the new lifestyle after giving birth by remembering the advantages of lifestyle change. Dencker et al., concluded that healthy diet and exercise during pregnancy did not persist after childbirth andprenatal care provided by midwives shifts to infant care after birth; so they are primary factors for discontinuity of changes [23]; in conclusion, pregnancy requires the support of the partner and then the relatives. However, the partner and relatives should keep in mind that the postpartum period is very important, both for the baby’s health and the woman's health. Therefore, the partner and relatives should continue to provide support after delivery.

On the other hand, health care providers should be aware that lifestyle changes in pregnancy, if not motivated to continue, return to pre-pregnancy status. Thus, by educating and counseling about the impact of maternal health on the present and future health of the family and children, the motivation for sustaining health-promoting behaviors should be strengthened for a long time or forever. Training partners to support women should always be on the agenda of health care providers.

### Implications for health policy and practice

Pregnant women are generally open to health-promoting changes in their lifestyle. Health care providers, including doctors, midwives, and other health care professionals, should utilize this opportunity to inform and motivate pregnant women on health-promoting lifestyle choices, including proper nutrition and sleep, activity during and after pregnancy, marital relationship, and reducing stress.

On the other hand, since pregnant mothers view social media as a main source of information, maternal health care providers should identify reputable websites to access accurate information and introduce them to mothers. Another important point is that partners should support their pregnant wives and be educated about the importance of support during pregnancy. Close relatives should be educated on the assistance they can provide for a pregnant woman to have a better pregnancy. Most importantly, pregnancy is seen an opportunity to change behaviors and adopt a healthy lifestyle. If lifestyle does not sustain after delivery, they may return to the pre-pregnancy period. The role of health care providers at this stage is very important. Women need to be aware that their health is significant to themselves and their families. Pregnancy care should continue in the postpartum period. Community-based care that is lacking in Iran can help continue care. Policy-making for community-based care is necessary. Husbands should receive routine care consultation, physical health tips on interpersonal interactions with the partner, and on how to provide mutual support.

Iranian women mentioned returning to work as an opportunity to care about themselves; organizational measures and policies should be designed to address maternal health and policies for more childcare, such as breastfeeding hours.

In the end, the results of the present study may be helpful for researchers for designing interventions that aim to sustain positive changes during and beyond pregnancy through family-centered approach empowerment, motivation, and behavioral goals.

### Limitations

The study faced some limitations. First, all participants received prenatal care. Therefore, present study results are limited to women who have already overcome the barrier of access to care, and our sample is less representative of women who are not in or delay their care. Moreover, information about lifestyle provided by health professionals may have changed ‘participants’ approach to how they answered interview questions, which may result in selection bias.

Another limitation is that multiparous women who talked about lifestyle changes during pregnancy and the unsustainability of the changes after delivery backed to 3-7 years ago, which may be a recall bias. Of course, clearly described experiences reduced the likelihood of bias.

All women in the study had a low-risk pregnancy. Repeating study on women with high-risk pregnancies can help expand the results.

Another limitation of the study was interviewing only pregnant and employed women. Interviewing the partners and relatives of the participants prand maternal health care providers can help clarify the issue from different aspects.

## Conclusion

The present qualitative study showed that pregnant women try to adopt health-promoting lifestyles and quit harmful behaviors due to the strong motive of childbearing. Lifestyle modifications are pervasive, encompassing all psychological, physical, and even religious aspects, but access to information and support from partners, relatives, friends, and health care providers, is very crucial in this regard. Women like to adhere to religious orders in pregnancy, where information is provided upon request. Unfortunately, many post-natal behaviors go back to the prenatal state, which is due to a lack of time and fatigue, reduced support of the partner and relatives, and reduced health care and education. Health care providers should keep caring about women during pregnancy and non-pregnancy to train healthy lifestyle and plan for the persistence of beneficial behaviors. Husbands should also be consulted about the importance of their supportive role in adopting and maintaining a healthy lifestyle in their wives.

## Data Availability

The dataset generated and/or analyzed during the present study is not publicly available but isavailable from the corresponding author on reasonable request.
